# Comparative Analysis of Chloroplast Genome Sequences and Phylogeny in Three *Macadamia integrifolia* Cultivars

**DOI:** 10.3390/genes16111248

**Published:** 2025-10-22

**Authors:** Jihua Guo, Zhuanmiao Kang, Zhongchun Xiao, Chunyan Zhong, Guidong Miao, Pei Zhang, Weiwei Zhao, Rongrong Su, Kecan Xia

**Affiliations:** 1 College of Biology and Chemistry, Minzu Normal University of Xingyi, Xingyi 562400, China; zhangpei@xynun.edu.cn (P.Z.); zhaoyuwei114@163.com (W.Z.); xiakecan@163.com (K.X.); 2 Guizhou Institute of Subtropical Crops, Guiyang 550025, China; 18085160451@163.com; 3 Key Laboratory of Biogenetic Resources Mining and Molecular Breeding in Qianxinan Prefecture, Xingyi 562400, Chinamiaoguidong@xynun.edu.cn (G.M.); 4 Biological Engineering Department, Southwest Guizhou Vocational and Technical College for Nationalities, Xingyi 562400, China; zhongchunyan0421@163.com; 5 Southwest Guizhou Institute of Agricultural and Forestry Sciences, Xingyi 562400, China; 18874076639@163.com

**Keywords:** *Macadamia integrifolia*, chloroplast genome, boundary analysis, phylogenetic analysis

## Abstract

**Background/Objectives**: *Macadamia integrifolia* is a valuable subtropical fruit tree, yet genomic studies on its cultivars are limited. This study aims to elucidate the chloroplast genome features, variations, and phylogenetic relationships of three main cultivars (‘Guilin No. 1’, ‘Nanya No. 1’, ‘Qian’ao No. 1’) to support germplasm identification and breeding. **Methods**: chloroplast genomes of three *M. integrifolia* cultivars from Guangxi, Guangdong, and Guizhou were sequenced using Illumina technology, followed by assembly, annotation, and comparative analyses of structure, repeats, and codon usage. Phylogenetic relationships were reconstructed using complete genome sequences. **Results**: The three chloroplast genomes displayed typical quadripartite structures, with lengths of 159,714 bp, 159,195 bp, and 159,508 bp, and GC contents of 38.12%, 38.16%, and 38.14%, respectively. Each encoded 135 genes. Codon usage was biased towards A/U-ending codons. We identified 81, 87, and 80 SSRs and 26, 21, and 20 long repeats, respectively. IR boundary regions were highly conserved. Phylogenetically, the cultivars showed close relationships with *M. integrifolia*, *Macadamia tetraphylla*, and *Macadamia* *ternifolia*, forming a sister clade to Platanus occidentalis. **Conclusions**: This study provides essential chloroplast genomic resources for three *M. integrifolia* cultivars, revealing conserved structures and specific variations. The findings offer crucial insights for the genus's genetic diversity, supporting future germplasm evaluation and phylogenetic research.

## 1. Introduction

*Macadamia integrifolia*, commonly known as the macadamia nut, is an evergreen tree belonging to the genus *Macadamia* in the family Proteaceae [1]. To date, four species of *Macadamia* have been identified: *M*. *integrifolia*, *Macadamia jansenii*, *M*. *ternifolia*, and *M*. *tetraphylla*. Their natural distribution is confined to the subtropical rainforests from southeastern Queensland to northeastern New South Wales, Australia [2,3]. Due to the superior quality of its fruit, which possesses nutritional, medicinal, and economic value, *M*. *integrifolia* is widely cultivated and traded internationally [4,5,6]. The species was first introduced to China in the 1970s. Currently, China has become the country with the largest planting area of *M*. *integrifolia* in the world, accounting for more than one-third of the global total, and remains the top producer worldwide [7].

Chloroplasts are vital organelles in green plants and algae, primarily responsible for photosynthesis, providing the essential energy source for their early growth and development [8,9]. As unique organelles to green plants, chloroplasts possess a complete semi-autonomous genetic system capable of semi-conservative replication. Their genetic material is termed the chloroplast genome [10,11]. The chloroplast genome is a closed circular DNA molecule with a highly conserved quadripartite structure comprising a large single-copy region (LSC), a small single-copy region (SSC), and two inverted repeat regions (IRa and IRb) [12,13]. Studies have shown that the chloroplast genomes of most angiosperms are predominantly maternally inherited and exhibit minimal genetic recombination [8,14]. Furthermore, compared with mitochondrial and nuclear genomes, chloroplast genomes are structurally conserved [15], possess high gene copy numbers [8], contain fewer repetitive sequences (excluding the conserved IR regions) [16], and show reduced rates of gene insertion, deletion, and mutation, coupled with moderate molecular evolutionary rates [14]. Recent studies have further refined the understanding of organellar DNA mutation patterns, suggesting that chloroplast genome stability is also regulated by DNA repair mechanisms [17]. It should be noted that the ‘fewer repetitive sequences’ refer to non-IR repetitive elements (such as SSRs and short tandem repeats). The large inverted repeat (IR) regions (typically 20–30 kb in angiosperms) are highly conserved duplicated segments that play a role in maintaining genome stability, which are not included in the ‘fewer repetitive sequences’ definition [18]. Consequently, chloroplast genomes have become essential tools in molecular ecology, synthetic biology, and crop breeding research that are widely applied in species identification, genetic diversity assessment, and phylogenetic analysis [19,20].

Existing studies have investigated the genetic characteristics of *M. integrifolia* from multiple dimensions. For instance, Lin et al. identified selective signals during domestication through whole-genome resequencing, revealing that positive selection on lipid synthesis-related genes (e.g., FAD2) played a key role in quality improvement [1]. Niu et al. assembled the mitochondrial genomes of three *M. integrifolia* species and uncovered potential influences of mitochondrial gene rearrangements on species adaptation [3]. In chloroplast genomics, Nock et al. completed the first full chloroplast genome sequencing of *M*. *integrifolia*, confirming its highly conserved genomic structure among early diverging eudicots [21]. Subsequent studies by Liu et al. respectively characterized the chloroplast genomes of *M. ternifolia* and *M. tetraphylla*, demonstrating only 50–100 bp length variations in the inverted repeat regions among species [22,23]. However, these studies either focused on nuclear genomic domestication signals or were confined to native species or materials from single geographic origins, leaving comparative analyses of chloroplast genomes across cultivated varieties from different Chinese production regions unexplored. To address this gap, this study selected three varieties of *M*. *integrifolia* with strong regional representativeness, covering the typical climate types of China’s core *M. integrifolia* cultivation areas (subtropical monsoon climate, plateau subtropical climate, and coastal subtropical climate). The selected varieties were Guilin No. 1 (primarily cultivated in the karst area of Guangxi), Nanya No. 1 (mainly grown in the coastal area of Guangdong), and Qian’ao No. 1 (bred in the plateau area of Guizhou) [24]. Their chloroplast genomes were sequenced, and bioinformatics methods were used to analyze and compare the genomic features, repetitive sequences, and phylogenetic relationships. The aim was to investigate the influence of different geographical environments on the chloroplast genome of *M*. *integrifolia*, providing a data foundation for its genetic breeding, conservation, and utilization.

## 2. Materials and Methods

### 2.1. Plant Materials

Healthy and fresh leaf samples of the three *M. integrifolia* cultivars (Guilin No. 1, Nanya No. 1, and Qian’ao No. 1) used in this study were collected from three experimental sites: the Guangxi South Subtropical Agricultural Sciences Research Institute in Longzhou County, Chongzuo City, Guangxi (22°33′49″ N, 106°79′20″ E), the South Subtropical Crop Research Institute, China Academy of Tropical Agricultural Sciences in Mazhang District, Zhanjiang City, Guangdong (21°16′96″ N, 110°27′08″ E), and Wanfenglin in Xingyi City, Guizhou Province (24°97′76″ N, 104°90′64″ E). Immediately after collection, the samples were flash-frozen in liquid nitrogen and stored at −80 °C in an ultra-low temperature freezer (Qingdao AuCMA Bio-Medical Co., Ltd. Shandong, China) for subsequent chloroplast genome sequencing and analysis.

### 2.2. DNA Extraction and Chloroplast Genome Sequencing

Total genomic DNA was extracted using a modified CTAB method (Chengdu Jinshan Chemical Reagent Co., Ltd., Chengdu, China). For each *M*. *integrifolia* cultivar, three healthy plants with consistent growth status were selected, and three fresh, pest-free leaves with uniform maturity were collected from each plant; leaves of the same cultivar were mixed as a sample (three biological replicates per cultivar). After qualifying the quality of the extracted DNA (detected by Nanodrop 2000 and 1% agarose gel electrophoresis (Beijing Liuyi Biotechnology Co., Ltd., Beijing, China; Sangon Biotech (Shanghai) Co., Ltd., Shanghai, China), OD260/OD280 = 1.8–2.0), the total high-quality DNA obtained from each cultivar was not less than 5 μg with a concentration ≥ 50 ng/μL. The samples were fragmented into approximately 350 bp fragments using an ultrasonic instrument (Shanghai Bilang Instrument Manufacturing Co., Ltd., Shanghai, China). The fragmented DNA then underwent purification, end repair, adenylation at the 3′ end, and adapter ligation. The products were size-selected via agarose gel electrophoresis (Beijing Liuyi Biotechnology Co., Ltd., Beijing, China; Sangon Biotech (Shanghai) Co., Ltd., Shanghai, China), amplified by PCR to form the sequencing library, and subjected to quality control. Once the library passed quality inspection, paired-end sequencing with a read length of 150 bp was performed on the Illumina (San Diego, CA, USA) NovaSeq platform.

### 2.3. Assembly and Annotation of Chloroplast Genomes

Following sequencing completion, raw data were filtered using FastQC v0.11.8 [25] to obtain high-quality clean data. The chloroplast genomes were then assembled using SPAdes v3.14.1 [26]. The complete chloroplast genome sequences were annotated for gene function with CPGAVAS2 v2 [27], and circular genome maps were drawn using OGDRAW v1.3 [28]. Finally, the annotated complete chloroplast genome sequences of the three *M. integrifolia* cultivars were submitted to NCBI to obtain the accession numbers (Guilin No. 1: PX289983; Nanya No. 1: PX289984; Qian’ao No. 1: PX289985).

### 2.4. Analysis of Chloroplast Genome Features

Protein-coding sequences were extracted from the genomes using Perl scripts. To ensure accuracy, duplicate sequences and those shorter than 300 bp were removed. Only sequences starting with ATG and ending with TAA, TAG, or TGA were selected. CodonW v1.4.4 software was used to analyze codon preference in protein-coding genes of the chloroplast genome and calculate RSCU values [28]. Additionally, MISA v1.0 software was employed for simple sequence repeat analysis, with parameters set as follows: 1–10 (mononucleotide repeats ≥ 10), 2-5, 3-4, 4-3, 5-3, 6-3 [29]. Long repeat sequences were analyzed using the online REPuter software (https://bibiserv.cebitec.uni-bielefeld.de/reputer, accessed on 18 October 2025), including forward, reverse, palindromic, and complementary repeats, with parameters set as: minimum length = 30 bp, Hamming distance = 3 [16]. The online program IRscope was used to visualize the IR/SC boundary regions and analyze expansion/contraction characteristics at the boundaries [30].

### 2.5. Phylogenetic Analysis

Twenty-one early diverging eudicot species were retrieved from GenBank as the ingroup, with the distantly related *Amborella trichopoda* serving as the outgroup. Using MEGA v12.0.9 software [31], the optimal evolutionary model (GTR + G + I) was selected via the ModelFinder function. Then, a phylogenetic tree was constructed based on the chloroplast genome sequences of these three *M*. *integrifolia* cultivars by the maximum likelihood (ML) method, with 1000 bootstrap replicates to evaluate node support, so as to further validate their phylogenetic positions.

## 3. Results

### 3.1. Structural Characteristics of the Chloroplast Genome

**Figure 1:**
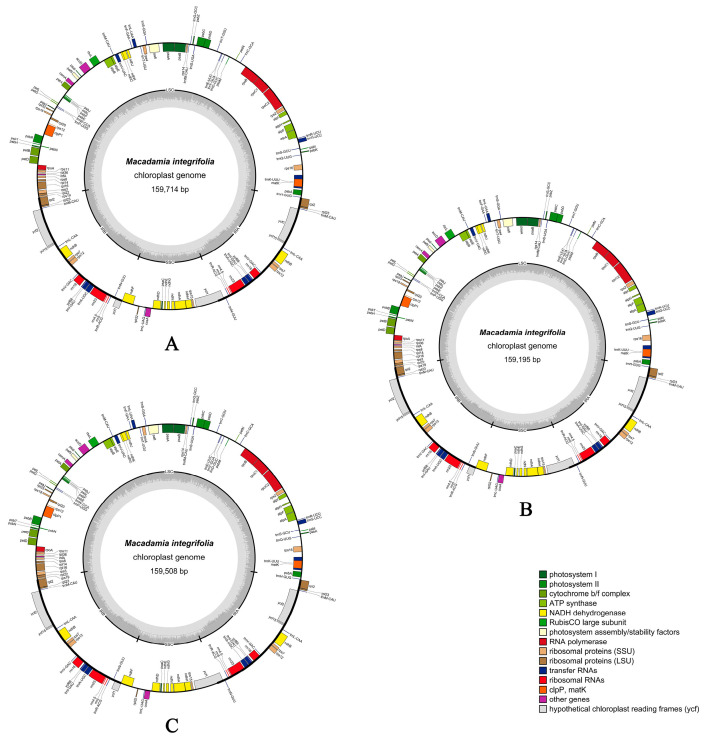
Chloroplast genome maps of the three *Macadamia integrifolia* cultivars. (**A**) Guilin No. 1, (**B**) Nanya No. 1, and (**C**) Qian’ao No. 1.

The chloroplast genomes of the three *M. integrifolia* cultivars (Guilin No. 1, Nanya No. 1, and Qian’ao No. 1) all exhibited a typical quadripartite circular structure. The complete chloroplast genome sequences were 159,714 bp, 159,195 bp, and 159,508 bp in length, respectively. The large single-copy (LSC) regions measured 88,093 bp, 87,651 bp, and 87,921 bp; the small single-copy (SSC) regions were 18,813 bp, 18,788 bp, and 18,743 bp; and the inverted repeat (IR) regions spanned 26,404 bp, 26,378 bp, and 26,422 bp. The overall GC contents were 38.12%, 38.16%, and 38.14%, respectively (Figure 1 and Table 1). The chloroplast genomes of the three *M*. *integrifolia* cultivars from different regions each encoded a total of 135 genes including 90 protein-coding genes (CDS), 37 tRNA genes, and 8 rRNA genes (Table 1). Based on their functions, these genes were categorized into four groups: photosynthesis-related genes, self-replication genes, biosynthesis-related genes, and genes with unknown functions (Table 2).

Among the annotated genes, all three cultivars contained 17 genes with introns, and the identity of these genes was consistent across the three samples. Specifically, six tRNA genes (*trnA-UGC*, *trnG-UCC*, *trnI-GAU*, *trnK-UUU*, *trnL-UAA*, *trnV-UAC*) and nine protein-coding genes (*atpF*, *ndhA*, *ndhB*, *petB*, *petD*, *rpl16*, *rpl2*, *rpoC1*, *rps16*) contained one intron each, while two protein-coding genes (*clpP1*, *pafI*) contained two introns each (Table 2). Additionally, thirteen protein-coding genes (*ndhB*, *rps12*, *rps7*, *rpl2*, *rpl23*, *rrn16*, *rrn23*, *rrn4.5*, *rrn5*, *ycf1*, *ycf15*, *ycf2*, *ycf68*) and six tRNA genes (*trnA-UGC*, *trnI-GAU*, *trnL-CAA*, *trnN-GUU*, *trnR-ACG*, *trnV-GAC*) were present in two copies each (Table 2).

### 3.2. Codon Preference Analysis

**Figure 2:**
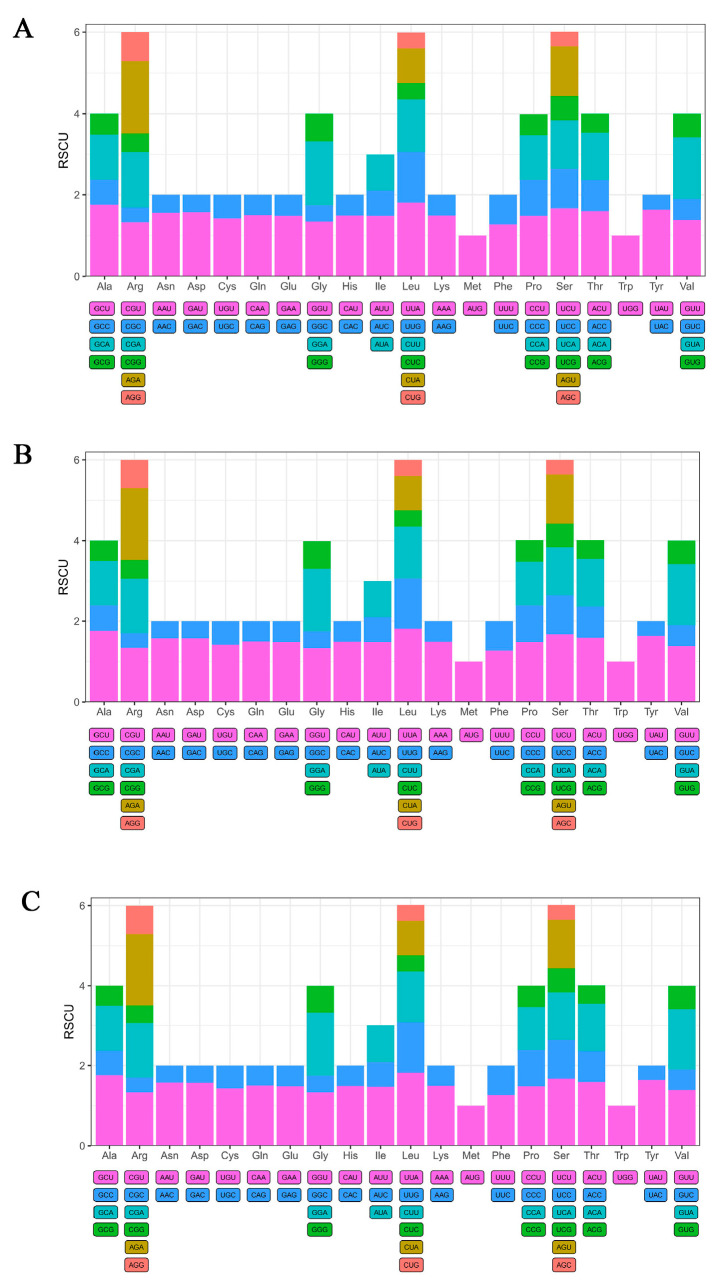
The relative usage degree of codons in the chloroplast genomes of three *Macadamia integrifolia* cultivars. (**A**) Guilin No. 1, (**B**) Nanya No. 1, and (**C**) Qian’ao No. 1.

The relative synonymous codon usage (RSCU) and codon preference of protein-coding genes in the chloroplast genomes were calculated and analyzed using CodonW software. The results indicated that the protein-coding genes in the chloroplast genomes of *M*. *integrifolia* Guilin No. 1, Nanya No. 1, and Qian’ao No. 1 were composed of 20,511, 20,512, and 20,503 codons, respectively. These codons were classified into 64 types encoding 20 amino acids. Among them, six codons encoded serine (Ser), leucine (Leu), and arginine (Arg); four codons encoded alanine (Ala), glycine (Gly), proline (Pro), and threonine (Thr); three codons encoded isoleucine (Ile); one codon each encoded methionine (Met) and tryptophan (Trp); and the remaining amino acids were encoded by two codons each (Table S1). Furthermore, the codon usage analysis revealed broadly similar patterns of codon preference across the three cultivars: 31 codons showed strong preference (RSCU > 1), of which 30 ended with A/U, with the exception of UUG; 31 codons exhibited weak preference (RSCU < 1), most of which ended with G/C except AUA and CUA; only two codons, AUG and UGG, showed no bias (RSCU = 1) (Figure 2). In this study, the highest RSCU value among the protein-coding genes in the three *M*. *integrifolia* cultivars’ chloroplast genomes was observed for UUA (encoding Leu), while the lowest values were recorded for AGC (encoding Ser) and CGC (encoding Arg).

### 3.3. Repeated Sequence Analysis

**Figure 3:**
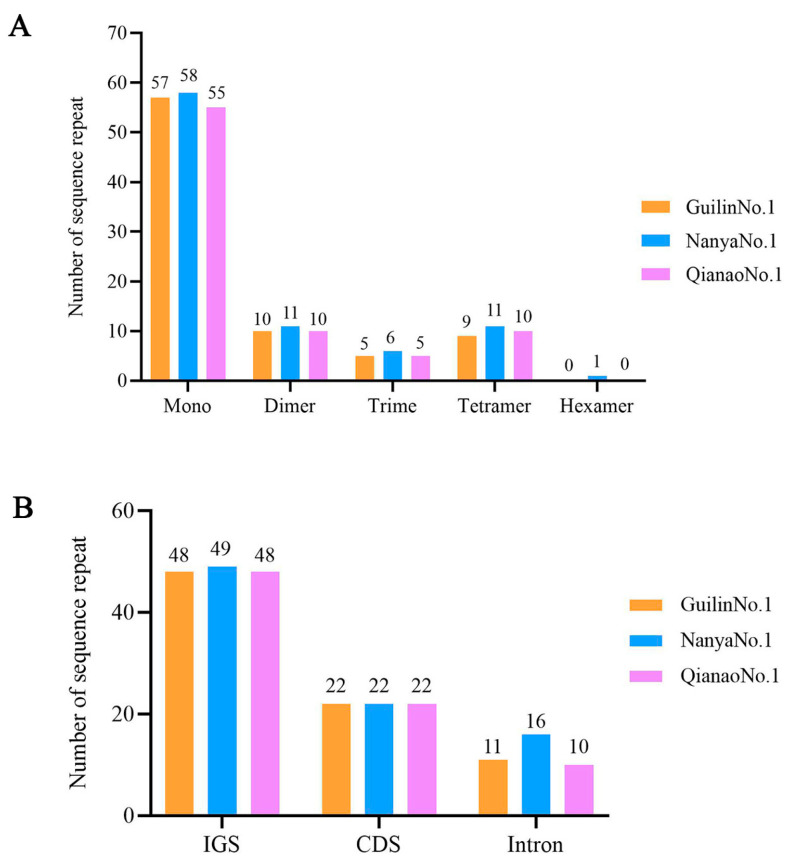
Classification and distribution positions of simple repetitive sequences (SSR) in the three *Macadamia integrifolia* cultivars. (**A**) Types of SSR. (**B**) Classification of SSR.

Simple sequence repeats (SSRs), a type of tandem repeat sequence typically no longer than 6 bp, are widely used in molecular marker development due to their dominant inheritance. In this study, a total of 81, 87, and 80 SSRs were detected in the chloroplast genomes of the three *M*. *integrifolia* cultivars (Guilin No. 1, Nanya No. 1, and Qian’ao No. 1), respectively. Among them, there were 57, 58, and 55 mononucleotides, 10, 11, and 10 dinucleotides, 5, 6, and 5 trinucleotides, and 9, 11, and 10 tetranucleotides, respectively. Additionally, a single hexanucleotide was identified in Nanya No.1 (Figure 3A and Table S2). Notably, SSRs in all three *M*. *integrifolia* cultivars were predominantly composed of mononucleotide repeats (A/T), followed by dinucleotide repeats (Figure 3A and Table S3). These SSRs were primarily concentrated in the LSC region, accounting for approximately 77.78% of the total, followed by the SSC region (14.81%), and finally the IR regions (7.40%). Regarding distribution locations, among the SSRs in the three *M*. *integrifolia* cultivars, 48, 49, and 48 were located in intergenic spacers (IGS), 22, 22, and 22 were in coding regions (CDS), and 11, 16, and 10 were in intron regions (Figure 3B).

**Figure 4:**
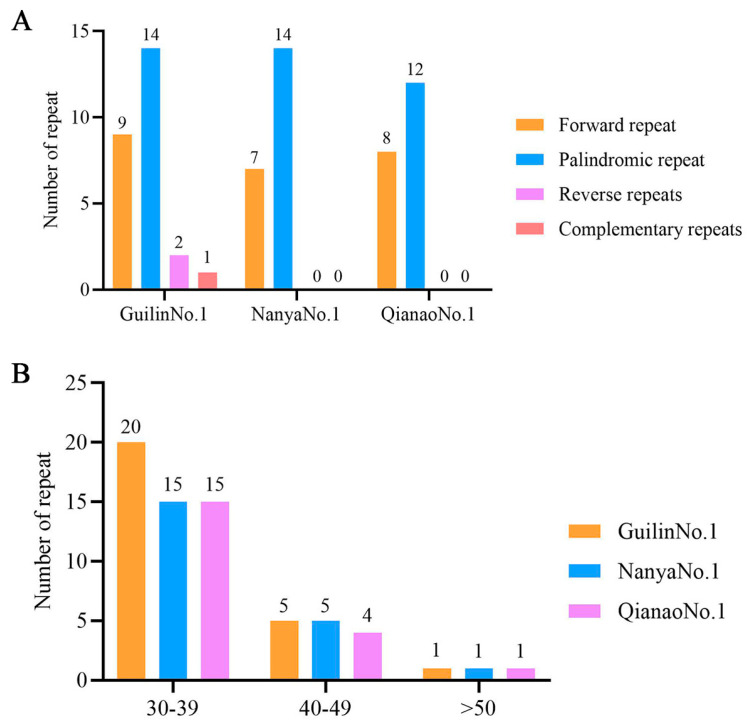
Analysis of the long repetitive sequences in the chloroplast genomes of three types of Australian nuts. (**A**) Total number of four types of repetitive sequences. (**B**) Length analysis of repetitive sequences.

Additionally, this study employed REPuter to analyze long repetitive sequences in the chloroplast genomes of the three *M*. *integrifolia* cultivars. The results revealed the presence of 26, 21, and 20 long repeat sequences in Guilin No. 1, Nanya No. 1, and Qian’ao No. 1, respectively. Among these, 9, 7, and 8 were forward repeats; 14, 14, and 12 were palindromic repeats; 2, 0, and 0 were reverse repeats; and 1, 0, and 0 were complementary repeats (Figure 4A). The length distribution of the repeat sequences predominantly ranged from 30 to 39 bp (Figure 4B), primarily located in the LSC and IR regions (Table S4).

### 3.4. IR Boundary Analysis

**Figure 5:**
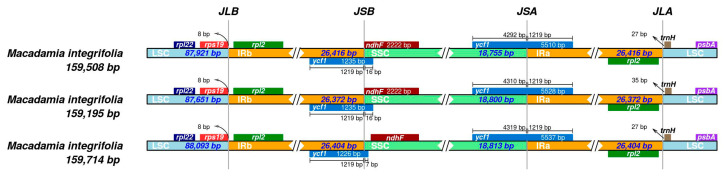
Analysis of the IR boundary changes in the chloroplast genomes of the three *Macadamia integrifolia* cultivars.

During the evolutionary process of plant chloroplast genomes, the contraction and expansion of IR boundaries are the primary factors determining size variations. This study analyzed the IR boundaries of the chloroplast genomes in the three *M*. *integrifolia* cultivars, with the results presented in Figure 5. The chloroplast genomes of these three *M*. *integrifolia* cultivars all possessed four boundaries: JLB (LSC/IRb), JSB (IRb/SSC), JSA (SSC/IRa), and JLA (IRa/LSC). The positions and types of these boundaries showed minimal variation among the three *M*. *integrifolia* cultivars’ chloroplast genomes. Specifically, the flanking genes at the JLB boundary were consistently *rpl22*, *rps19*, and *rpl2* in all three samples, while the JLA boundary was flanked by *rpl2* and *trnH*. At the JSB boundary, *ycf1* and *ndhF* were identified in all cultivars. The *ycf1* gene measured 1235 bp in both Guilin No. 1 and Nanya No. 1, and 1226 bp in Guilin No. 1. In all three cases, this gene exhibited a 1219 bp expansion into the IRb region. The JSA boundary was located within the coding region of the *ycf1* gene, which had total lengths of 5510 bp, 5528 bp, and 5537 bp in the three cultivars, respectively. Each also showed a 1219 bp expansion into the IRa region.

### 3.5. Phylogenetic Analysis of Three Macadamia integrifolia

**Figure 6:**
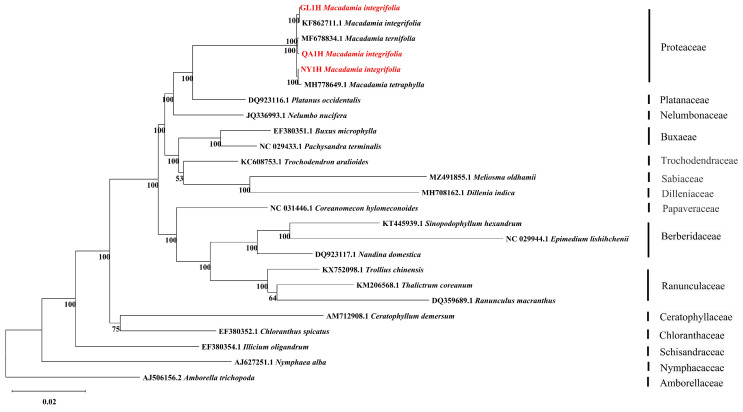
A ML phylogenetic tree of 25 species constructed based on the chloroplast genome using MEGA12. The numbers above each node represent the maximum likelihood support values (bootstrap replicates = 1000). The red markings indicate the three *Macadamia integrifolia* in this study.

A phylogenetic tree was constructed using maximum likelihood (ML) methods, with 24 typical early diverging eudicots as the inner group and *Amborella trichopod* from the Amborellaceae as the outgroup (Figure 6). The results indicated that the three *M*. *integrifolia* cultivars (Guilin No. 1, Nanya No. 1, and Qian’ao No. 1) were closely related to *M*. *integrifolia*, *M*. *tetraphylla*, and *M*. *ternifolia*, respectively. These six species clustered into a small branch within Proteaceae and formed a sister clade with *Platanus occidentalis* from the Platanaceae family at 100% support.

## 4. Discussion

This study collected *M*. *integrifolia* materials cultivated in the Guangxi, Guangdong, and Guizhou Provinces of China, and completed the assembly and comparative analysis of their complete chloroplast genomes. Compared with previous research, this study is the first to demonstrate that geographic and climatic divergence does not disrupt the conservation of the *M. integrifolia* chloroplast genome, thereby providing molecular evidence for the genetic stability of its cross-regional introduction and enriching the genetic data on this species at the population or geographical variation level.

Regarding genomic basic features, the chloroplast genomes of the three examined samples (Guilin No. 1, Nanya No. 1, and Qian’ao No. 1) were 159,714 bp, 159,195 bp, and 159,508 bp in length, each encoding 135 genes, with the total GC contents ranging from 38.12% to 38.16%. These values closely resemble those reported by Nock et al. for *M*. *integrifolia* and by Liu et al. for *M*. *ternifolia*, further supporting the high conservation of chloroplast genome size, structure, and gene content within the Proteaceae family [21,22]. These findings align with the conclusion proposed by Nock et al. that early diverging eudicot chloroplast genomes exhibit strong evolutionary conservation [15,21,32]. Analysis of codon usage bias revealed that all three *M*. *integrifolia* cultivars exhibited a significant preference for codons ending in A/U. This trend aligns with the general pattern observed in most angiosperm chloroplast genomes, likely resulting from the combined effects of mutation pressure and natural selection [33,34,35]. Compared with the study on *Macadamia tetraphylla* by Liu et al., this research further reveals consistency in codon usage patterns among geographic accessions, suggesting stable evolutionary pressures on their coding sequences [23].

In terms of repetitive sequences, SSRs in the three *M*. *integrifolia* cultivars were dominated by mononucleotide A/T repeats and were primarily distributed in intergenic spacer regions. This is similar to the findings of Hu et al. in *Nelumbo nucifera*, reaffirming the distribution bias of chloroplast SSRs [36]. Among the long repeats, palindromic and forward repeats were most abundant, consistent with the analysis of *M*. *integrifolia* by Nock et al. [21]. Notably, only Guilin No. 1 exhibited reverse and complementary repeats. These repetitive sequences were located in the intergenic spacer (IGS) between *trnG-UCC* (a tRNA-coding gene) and *psbZ* (a photosystem II gene). We hypothesize that they may function through the following mechanisms. (1) Regulation of adjacent gene expression [37]: the repeats may alter the secondary structure of the IGS region, influencing RNA polymerase binding efficiency and consequently enhancing the transcription levels of *trnG-UCC* and *psbZ*. This elevated expression could improve the photosynthetic efficiency, potentially serving as an adaptation to the high-light and nutrient-poor conditions of the karst region in Guangxi. (2) Involvement in DNA damage repair [38]: the inverted repeats may facilitate the repair of DNA breaks induced by high-light stress through homologous recombination, thereby contributing to the maintenance of chloroplast genome stability. Future validation will require quantifying the expression levels of these genes via qPCR and measuring relevant photosynthetic parameters to test these hypotheses.

IR boundary analysis revealed that the four boundary regions and their flanking genes were highly conserved across all samples, with no significant expansion or contraction observed. This contrasts with findings by Li et al. in *Camellia* species., where noticeable IR boundary shifts were detected among different species [39]. Our results indicate extremely high IR region stability within *M. integrifolia*, with no major structural variations attributable to geographical differences. Phylogenetic analysis revealed that the three *M. integrifolia* cultivars (‘Guilin 1’, ‘Nanya 1’, and ‘Qian’ao 1’) exhibited close phylogenetic relationships with *M. integrifolia*, *M. tetraphylla*, and *M. ternifolia*, respectively. This finding is consistent with the documented breeding backgrounds of the cultivars: the maternal parent of ‘Guilin 1’ is *M. integrifolia*; ‘Nanya 1’ possesses paternal ancestry from *M. tetraphylla*; and ‘Qian’ao 1’ was developed through selective breeding from a hybrid between *M. ternifolia* and a local species. The clustering pattern, where each cultivar groups with its corresponding maternal progenitor, can be attributed to the maternal inheritance of the chloroplast genome [40]. Furthermore, phylogenetic analysis strongly supported (100% bootstrap value) the interspecific relationships within *Macadamia* and its sister-group relationship with *Platanus occidentalis*. This is highly consistent with the phylogenies proposed by Nock et al. and Mast et al. based on chloroplast and nuclear gene data, further consolidating the phylogenetic position of Proteaceae within basal eudicots [2,21].

Through a comparative analysis of three *M. integrifolia* chloroplast genomes, we demonstrated high conservation in the genome structure, gene content, and GC content, supporting the hypothesis of “evolutionary inertia” in early diverging eudicots (e.g., Proteaceae) [15,32]. The slow evolutionary rate of chloroplast genomes in these species may be associated with their woody perennial life history (characterized by long generation times and slow mutation accumulation) and efficient DNA repair mechanisms [41,42]. Moreover, the unique repeat sequences identified in the LSC intergenic spacer of ‘Guilin 1’ suggest that even within a conserved genomic background, non-coding regions can accumulate genetic diversity through small-scale structural variations (e.g., inverted and complementary repeats). These regions may thereby serve as “innovation hotspots” for adaptive evolution, consistent with the general paradigm of chloroplast genome evolution wherein coding regions remain highly conserved while non-coding regions exhibit structural plasticity [43]. The conserved nature of the chloroplast genome makes it an ideal marker for germplasm identification in woody crops [44]. The three cultivar-specific SSRs identified in this study (e.g., the mononucleotide repeat A10 in ‘Guilin 1’ and the hexanucleotide repeat in ‘Nanya 1’) can be developed as molecular markers to address cultivar admixture issues during *M. integrifolia* seedling propagation. For instance, the A10 marker enables the rapid PCR-based differentiation of ‘Guilin 1’ from other cultivars. Furthermore, the maternally inherited characteristics of the chloroplast can be utilized to trace genealogical relationships among cultivars [45]. By conducting sequence alignment of the chloroplast *rbcL* gene, the genetic similarity between ‘Qian’ao 1’ and wild Australian *M. integrifolia* can be clarified, thereby providing molecular evidence for historical tracing of introduction and domestication processes.

In summary, although the samples originated from three regions with distinct climatic and environmental conditions, their chloroplast genomes exhibited high consistency. Recent advances in pan-plastome analysis have provided new insights into the genetic diversity of crop chloroplast genomes. For example, the pan-plastome of Pisum sativum revealed that non-core genes (e.g., toxin-related genes) contributed significantly to intraspecific variation, while core genes (e.g., photosynthesis-related genes) remained highly conserved [46]. This is consistent with our findings: the three *M. integrifolia* cultivars shared 135 core chloroplast genes (all involved in photosynthesis and self-replication), with no non-core genes detected, indicating that the chloroplast genome of *M. integrifolia* may have a relatively small pan-genome size. Future studies could construct a pan-plastome of *M. integrifolia* by including more wild relatives (e.g., *M. jansenii*) and landraces, which would help identify rare genetic variations and their potential roles in environmental adaptation. It is noteworthy that although the samples in this study covered the main cultivars from China’s major production regions, they did not include wild resources or introduced foreign cultivars, which may not fully capture the entire genetic diversity of the *Macadamia*. Future studies could incorporate wild populations from the native origin of *M. integrifolia* (Australia) and distinctive cultivars from production areas such as Yunnan and Hainan in China to further validate the universality of chloroplast genome conservation. Meanwhile, integrating nuclear genome SNP data could provide a more comprehensive analysis of the genetic differentiation patterns in *Macadamia*.

## 5. Conclusions

This study conducted the first comparative analysis of chloroplast genomes from three geographically distinct sources of *M*. *integrifolia*, revealing high similarity in genomic structure, codon usage preferences, and repetitive sequence distribution. This further confirms the high conservation of chloroplast genomes within this species. Phylogenetic analysis also supports a sister relationship between the Proteaceae and Platanaceae families. This establishes a data foundation for genetic breeding, systematics, and functional genomics research on macadamia nuts.

## Figures and Tables

**Table 1:** Basic characteristics of the chloroplast genomes of the three *Macadamia integrifolia*.

Species	Guilin No. 1	Nanya No. 1	Qianao No. 1
Total length (bp)	159,714	159,195	159,508
LSC. length (bp)	88,093	87,651	87,921
SSC. length (bp)	18,813	18,788	18,743
IR. length (bp)	26,404	26,378	26,422
Total GC (%)	38.12%	38.16%	38.14%
Genes number	135	135	135
Protein-coding genes	90	90	90
tRNA genes	37	37	37
rRNA genes	8	8	8

**Table 2:** Common genes annotated in the chloroplast genomes of the three *Macadamia integrifolia*.

Category of Genes	Group of Genes	Name of Genes	Number
Photosynthesis	Photosystem I	*psaA*, *psaB*, *psaC*, *psaI*, *psaJ*, *pafI* **, *pafII*	7
	Photosystem II	*psbA*, *psbB*, *psbC*, *psbD*, *psbE*, *psbF*, *psbH*, *psbI*, *psbJ*, *psbK*, *psbL*, *psbM*, *psbN*, *psbT*, *psbZ*	15
	NADH-dehydrogenase	*ndhA* *, *ndhB*(x2) *, *ndhC*, *ndhD*, *ndhE*, *ndhF*, *ndhG*, *ndhH*, *ndhI*, *ndhJ*, *ndhK*	12
	Cytochrome b/6f complex	*petA*, *petB* *, *petD* *, *petG*, *petL*, *petN*	6
	ATP synthase	*atpA*, *atpB*, *atpE*, *atpF* *, *atpH*, *atpI*	6
	Rubisco	*rbcL*	1
Self-replication	Large subunit of ribosome	*rpl14*, *rpl16* *, *rpl2*(x2) *, *rpl20*, *rpl22*, *rpl23*(x2), *rpl32*, *rpl33*, *rpl36*	11
	Small subunit of ribosome	*rps11*, *rps12*(x2), *rps14*, *rps15*, *rps16* *, *rps18*, *rps19*, *rps2*, *rps3*, *rps4*, *rps7*(x2), *rps8*	14
	DNA dependent RNA polymerase	*rpoA*, *rpoB*, *rpoC1* *, *rpoC2*	4
	rRNA genes	*rrn16*(x2), *rrn23*(x2), *rrn4.5*(x2), *rrn5*(x2)	8
	tRNA genes	*trnA-UGC*(x2) *, *trnC-GCA*, *trnD-GUC*, *trnE-UUC*, *trnF-GAA*, *trnG-GCC*, *trnG-UCC* *, *trnH-GUG*, *trnI-GAU*(x2) *, *trnK-UUU* *, *trnL-CAA*(x2), *trnL-UAA* *, *trnL-UAG*, *trnM-CAU*(x3), *trnN-GUU*(x2), *trnP-UGG*, *trnQ-UUG*, *trnR-ACG*(x2), *trnR-UCU*, *trnS-GCU*, *trnS-GGA*, *trnS-UGA*, *trnT-GGU*, *trnT-UGU*, *trnV-GAC*(x2), *trnV-UAC* *, *trnW-CCA*, *trnY-GUA*, *trnfM-CAU*	37
Biosynthesis	Maturase	*matK*	1
	Protease	*clpP1* **	1
	Envelope membrane protein	*cemA*	1
	Acetyl-CoA carboxylase	*accD*	1
	C-type cytochrome synthesis gene	*ccsA*	1
	Translation initiation factor	*infA*	1
Unknown function	Conserved hypothetical chloroplast reading frames	*ycf1*(x2), *ycf15*(x2), *ycf2*(x2), *ycf68*(x2)	8

Note: * indicates genes containing introns, ** indicates having two introns, and (x2) indicates that the gene sequence was repeated twice.

## Data Availability

The original contributions presented in this study are included in the article/Supplementary Materials. Further inquiries can be directed to the corresponding author.

## Supplementary Materials

The following supporting information can be downloaded at: https://www.mdpi.com/article/10.3390/genes16111248/s1, Table S1. Relative Synonymous Codon Usage (RSCU) of *Macadamia integrifolia* CP genomes; Table S2. Number of different SSR types detected in three *Macadamia integrifolia*; Table S3. Frequency of identified SSR motifs in different repeat class types; Table S4 Statistical of number of long repeat sequence types.
