# Supplementary material for: Comparative Analysis of Chloroplast Genome Sequences and Phylogeny in Three Macadamia integrifolia Cultivars
Source: Genes (Basel). 2025 Oct 22;16(11):1248. doi: 10.3390/genes16111248 (PMC12652687; doi:10.3390/genes16111248)
Supplement: Supplementary file 1 [file genes-16-01248-s001.zip › genes-3917028-supplementary.pdf]

Table S1. Relative Synonymous Codon Usage (RSCU) of *Macadamia integrifolia* CP genomes

Species	Amino acid	codon	Number	RSCU	Amino acid	codon	Number	RSCU
Guilin No. 1	AGG	Arg	147	0.71	AUG	Met	474	1
	GUA	Val	426	1.52	CCG	Pro	112	0.52
	UUG	Leu	440	1.26	GAA	Glu	844	1.48
	AAG	Lys	259	0.51	UGG	Trp	381	1
	UGA	end	19	1.14	GUU	Val	385	1.38
	CCA	Pro	232	1.09	CAC	His	128	0.51
	GAG	Glu	295	0.52	UCC	Ser	253	0.98
	UAA	end	22	1.32	CGC	Arg	75	0.36
	AUA	Ile	524	0.9	UGU	Cys	167	1.42
	GGG	Gly	240	0.68	CCU	Pro	317	1.48
	GCU	Ala	500	1.76	UAU	Tyr	627	1.63
	AGC	Ser	93	0.36	AUU	Ile	856	1.48
	UUC	Phe	418	0.73	GCA	Ala	316	1.11
	AAC	Asn	216	0.44	CAG	Gln	184	0.5
	GAC	Asp	184	0.43	CGG	Arg	94	0.45
	CUU	Leu	447	1.28	UCG	Ser	152	0.59
	GGC	Gly	147	0.41	CUA	Leu	298	0.85
	ACU	Thr	414	1.6	ACA	Thr	307	1.18
	UCU	Ser	434	1.67	UCA	Ser	308	1.19
	CGU	Arg	277	1.33	CGA	Arg	284	1.37
	CAU	His	373	1.49	GCG	Ala	144	0.51
	GUC	Val	145	0.52	CAA	Gln	556	1.5
	UAC	Tyr	140	0.37	ACG	Thr	120	0.46
	AUC	Ile	358	0.62	CUG	Leu	138	0.39
	UGC	Cys	68	0.58	UUU	Phe	727	1.27
	CCC	Pro	193	0.9	AAU	Asn	777	1.56
	UUA	Leu	634	1.81	GCC	Ala	175	0.62
	AAA	Lys	766	1.49	AGU	Ser	316	1.22
	GUG	Val	163	0.58	GGU	Gly	473	1.34
	AGA	Arg	371	1.78	ACC	Thr	197	0.76
	GGA	Gly	557	1.57	GAU	Asp	674	1.57
	UAG	end	9	0.54	CUC	Leu	141	0.4
Nanya No. 1	UAC	Tyr	141	0.37	UCC	Ser	251	0.97
	CAC	His	127	0.51	CCC	Pro	194	0.91
	UCU	Ser	434	1.67	UAU	Tyr	629	1.63
	CCU	Pro	316	1.48	CAU	His	373	1.49
	CCA	Pro	233	1.09	UAA	end	22	1.32
	ACG	Thr	120	0.46	AAG	Lys	260	0.51
	UCA	Ser	311	1.2	CAA	Gln	556	1.5

	AGU	Ser	316	1.22	AGC	Ser	93	0.36
	UGG	Trp	380	1	GUG	Val	164	0.58
	AGA	Arg	368	1.78	GUA	Val	426	1.52
	CGG	Arg	95	0.46	GUU	Val	388	1.38
	UGA	end	19	1.14	CGC	Arg	75	0.36
	AGG	Arg	146	0.7	UGC	Cys	67	0.58
	CGA	Arg	282	1.36	UAG	end	9	0.54
	GUC	Val	146	0.52	CAG	Gln	185	0.5
	UGU	Cys	166	1.42	AAA	Lys	769	1.49
	CGU	Arg	277	1.34	AAU	Asn	776	1.57
	ACA	Thr	308	1.19	ACC	Thr	199	0.77
	CCG	Pro	113	0.53	GAA	Glu	845	1.48
	UCG	Ser	151	0.58	GCC	Ala	178	0.63
	ACU	Thr	412	1.59	GAU	Asp	674	1.57
	AAC	Asn	212	0.43	UUG	Leu	439	1.26
	GCA	Ala	316	1.11	AUA	Ile	522	0.9
	GAC	Asp	184	0.43	CUG	Leu	139	0.4
	GCU	Ala	498	1.76	AUU	Ile	854	1.48
	GGG	Gly	243	0.68	GGC	Gly	150	0.42
	AUC	Ile	360	0.62	UUU	Phe	727	1.27
	UUC	Phe	417	0.73	CUU	Leu	447	1.28
	CUC	Leu	140	0.4	UUA	Leu	634	1.81
	GGU	Gly	472	1.33	CUA	Leu	297	0.85
	GGA	Gly	555	1.56	AUG	Met	475	1
	GCG	Ala	142	0.5	GAG	Glu	295	0.52
Qianao No. 1	CAC	His	127	0.51	UUA	Leu	635	1.82
	ACG	Thr	120	0.46	ACU	Thr	412	1.59
	CCA	Pro	231	1.08	UAU	Tyr	628	1.64
	UAG	end	9	0.54	GAC	Asp	184	0.43
	ACC	Thr	199	0.77	AGC	Ser	93	0.36
	UAC	Tyr	139	0.36	AUC	Ile	359	0.62
	GUA	Val	427	1.52	GCA	Ala	319	1.13
	AGU	Ser	317	1.22	UGA	end	19	1.14
	GAU	Asp	675	1.57	CUA	Leu	300	0.86
	CAG	Gln	184	0.5	AUG	Met	474	1
	UCA	Ser	311	1.2	CAU	His	375	1.49
	AAA	Lys	765	1.5	GAG	Glu	294	0.52
	GGA	Gly	554	1.57	CGA	Arg	283	1.36
	AUU	Ile	855	1.47	AGG	Arg	147	0.71
	AAG	Lys	258	0.5	UUC	Phe	415	0.73
	CAA	Gln	555	1.5	GAA	Glu	845	1.48
	UCG	Ser	153	0.59	AGA	Arg	370	1.78
	CUU	Leu	446	1.28	CGG	Arg	94	0.45

GGG	Gly	241	0.68	GUU	Val	390	1.39
CCC	Pro	194	0.91	AUA	Ile	525	0.91
GUG	Val	163	0.58	CUG	Leu	137	0.39
CGU	Arg	276	1.33	GGU	Gly	471	1.33
CCG	Pro	114	0.53	AAU	Asn	774	1.57
UAA	end	22	1.32	UCU	Ser	433	1.67
ACA	Thr	308	1.19	GCC	Ala	174	0.61
UUU	Phe	725	1.27	UGC	Cys	67	0.57
GUC	Val	144	0.51	CUC	Leu	140	0.4
AAC	Asn	212	0.43	GCG	Ala	142	0.5
UCC	Ser	252	0.97	UGG	Trp	380	1
GGC	Gly	149	0.42	UUG	Leu	440	1.26
UGU	Cys	168	1.43	CCU	Pro	316	1.48
GCU	Ala	499	1.76	CGC	Arg	76	0.37

Table S2. Number of different SSR types detected in three *Macadamia integrifolia*

Type	Composition	Number		
		Guilin No. 1	Nanya No. 1	Qianao No. 1
Mono	A	27	28	27
	T	30	30	28
Dimer	AT	5	4	6
	CT	1	1	1
	TA	3	5	2
	TC	1	1	1
Trime	ATA	2	2	2
	ATG	1	1	1
	TAA	1	1	1
	TTA	1	2	1
Tetramer	AATA	1	1	1
	AATC	1	1	1
	AATT	1	1	1
	CATT	1	1	1
	TAAA	2	1	3
	TATT	1	3	1
	TCAA	1	1	1
	TTCT	1	1	1
Hexamer	TAAAAA	0	1	0
Total		81	87	80

Table S3. Frequency of identified SSR motifs in different repeat class types

Type	Number		
	Guilin No. 1	Nanya No. 1	Qianao No. 1
A/T	57	58	55
AG/CT	2	2	2
AT/AT	8	9	8
AAT/ATT	4	5	4
ATC/ATG	1	1	1
AAAG/CTTT	1	1	1
AAAT/ATTT	4	5	5
AATC/ATTG	2	2	2
AATG/ATTC	1	1	1
AATT/AATT	1	2	1
AAAAAT/ATTTTT	0	1	0

Table S4 Statistical of number of long repeat sequence types

Species	Length	Type	Gene1	Gene2	Region1	Region2
Guilin No. 1	26404	P	*ycf1*	*rps19, rpl2*	IRB	IRA
	42	P	*trnE-UUC, trnT-GGU*	*trnE-UUC, trnT-GGU*	LSC	LSC
	38	F	*trnR-UCU, atpA*	*trnR-UCU, atpA*	LSC	LSC
	42	F	*pafI-intron*	*rps12, trnV-GAC*	LSC	IRB
	42	P	*pafI-intron*	*trnV-GAC_rps12*	LSC	IRA
	34	P	*psbI, trnS-GCU*	*psbI, trnS-GCU*	LSC	LSC
	43	P	*trnS-GCU, trnG-UCC*	*trnS-GCU, trnG-UCC*	LSC	LSC
	40	F	*psaB*	*psaA*	LSC	LSC
	32	P	*petA_psbJ*	*petA_psbJ*	LSC	LSC
	30	P	*trnS-GGA*	*psbI, trnS-GCU*	LSC	LSC
	34	F	*psbI, trnS-GCU*	*psbC, trnS-UGA*	LSC	LSC
	33	R	*trnT-GGU, psbD*	*trnT-GGU, psbD*	LSC	LSC
	31	C	*trnK-UUU, rps16*	*ndhF, rpl32*	LSC	SSC
	31	P	*rps8, rpl14*	*rps8, rpl14*	LSC	LSC
	31	F	*ycf2*	*ycf2*	IRB	IRB
	31	P	*ycf2*	*ycf2*	IRB	IRA
	31	P	*ycf2*	*ycf2*	IRB	IRA
	31	P	*trnR-ACG, trnN-GUU*	*trnR-ACG, trnN-GUU*	IRB	IRB

	31	F	*trnR-ACG, trnN-GUU*	*trnN-GUU, trnR-ACG*	IRB	IRA
	31	P	*trnN-GUU, trnR-ACG*	*trnN-GUU, trnR-ACG*	IRA	IRA
	31	F	*ycf2*	*ycf2*	IRA	IRA
	30	P	*trnT-GGU, psbD*	*trnT-GGU, psbD*	LSC	LSC
	30	F	*psbZ, trnG-GCC*	*pafI-intron*	LSC	LSC
	30	F	*pafI-intron*	*rps12, trnV-GAC*	LSC	IRB
	30	P	*pafI-intron*	*trnV-GAC, rps12*	LSC	IRA
	30	R	*trnT-UGU, trnL-UAA*	*pafII, cemA*	LSC	LSC
Nanya No. 1	26372	P	*ycf1*	*rps19, rpl2*	IRB	IRA
	42	P	*trnE-UUC, trnT-GGU*	*trnE-UUC, trnT-GGU*	LSC	LSC
	42	F	*pafI-intron*	*rps12, trnV-GAC*	LSC	IRB
	42	P	*pafI-intron*	*trnV-GAC, rps12*	LSC	IRA
	43	P	*trnS-GCU, trnG-UCC*	*trnS-GCU, trnG-UCC*	LSC	LSC
	40	F	*psaB*	*psaA*	LSC	LSC
	32	P	*petA, psbJ*	*petA, psbJ*	LSC	LSC
	32	P	*petA, psbJ*	*petA, psbJ*	LSC	LSC
	30	P	*trnS-GGA*	*psbI_trnS-GCU*	LSC	LSC
	34	F	*psbI, trnS-GCU*	*psbC, trnS-UGA*	LSC	LSC
	31	P	*trnT-GGU, psbD*	*trnT-GGU, psbD*	LSC	LSC
	31	P	*rps8, rpl14*	*rps8, rpl14*	LSC	LSC
	31	F	*ycf2*	*ycf2*	IRB	IRB
	31	P	*ycf2*	*ycf2*	IRB	IRA
	31	P	*ycf2*	*ycf2*	IRB	IRA
	31	P	*trnR-ACG, trnN-GUU*	*trnR-ACG, trnN-GUU*	IRB	IRB
	31	F	*trnR-ACG, trnN-GUU*	*trnN-GUU, trnR-ACG*	IRB	IRA
	31	P	*trnN-GUU, trnR-ACG*	*trnN-GUU, trnR-ACG*	IRA	IRA
	31	F	*ycf2*	*ycf2*	IRA	IRA
	30	F	*pafI-intron*	*rps12, trnV-GAC*	LSC	IRB
	30	P	*pafI-intron*	*trnV-GAC, rps12*	LSC	IRA
Qianao No. 1	26416	P	*ycf1*	*rps19, rpl2*	IRB	IRA
	42	P	*trnE-UUC, trnT-GGU*	*trnE-UUC, trnT-GGU*	LSC	LSC
	42	F	*pafI-intron*	*rps12, trnV-GAC*	LSC	IRB
	42	P	*pafI-intron*	*trnV-GAC, rps12*	LSC	IRA

34	P	*psbI, trnS-GCU*	*psbI, trnS-GCU*	LSC	LSC
40	F	*psaB*	*psaA*	LSC	LSC
30	F	*rpl33, rps18*	*rpl33, rps18*	LSC	LSC
32	P	*petA, psbJ*	*petA, psbJ*	LSC	LSC
30	P	*trnS-GGA*	*psbI_trnS-GCU*	LSC	LSC
34	F	*psbI, trnS-GCU*	*psbC, trnS-UGA*	LSC	LSC
31	P	*rps8, rpl14*	*rps8, rpl14*	LSC	LSC
31	F	*ycf2*	*ycf2*	IRB	IRB
31	P	*ycf2*	*ycf2*	IRB	IRA
31	P	*ycf2*	*ycf2*	IRB	IRA
31	P	*trnR-ACG, trnN-GUU*	*trnR-ACG, trnN-GUU*	IRB	IRB
31	F	*trnR-ACG, trnN-GUU*	*trnN-GUU, trnR-ACG*	IRB	IRA
31	P	*trnN-GUU, trnR-ACG*	*trnN-GUU, trnR-ACG*	IRA	IRA
31	F	*ycf2*	*ycf2*	IRA	IRA
30	F	*pafI-intron*	*rps12, trnV-GAC*	LSC	IRB
30	P	*pafI-intron*	*trnV-GAC, rps12*	LSC	IRA
